# Exercise in an Overweight Patient with Covid-19: A Case Study

**DOI:** 10.3390/ijerph18115882

**Published:** 2021-05-30

**Authors:** Amir Hossein Ahmadi Hekmatikar, Mahdieh Molanouri Shamsi, Zahra Sadat Zabhi Ashkazari, Katsuhiko Suzuki

**Affiliations:** 1 Department of Physical Education & Sport Sciences, Faculty of Humanities, Tarbiat Modares University, Tehran 14117-13116, Iran; a.ahmadihekmatik@modares.ac.ir; 2 Department of Infectious Diseases, School of Medicine, Iran University of Medical Sciences, Tehran 14496-14535, Iran; zebhi.zahra@gmail.com; 3 Faculty of Sport Sciences, Waseda University, 2-579-15, Mikajima, Tokorozawa 359-1192, Japan

**Keywords:** coronavirus, exercise, patient with COVID-19, training, SARS-CoV-2

## Abstract

Coronavirus (COVID-19) is a dangerous infectious disease that is easily transmitted and which is called an acute respiratory syndrome. With the spread of the coronavirus around the world and its epidemic among humans, we are losing many humans. The long process of treatment in hospitalized patients who are receiving intensive care and medication is associated with physical weakness. It has been suggested that lifelong exercise can create a safe margin for a person that allows them to avoid becoming infected with the virus. The current study was conducted to assess the effects of low-intensity exercise and breathing exercises on cardiorespiratory responses and physical status in an overweight 20-year-old woman infected with COVID-19. The patient was referred to Hazrat Ali Ibn Abitaleb Hospital in Rafsanjan. The patient had initial symptoms of coronavirus including weakness, shortness of breath, fever, and chills, and the initial tests confirmed that the person was infected with the coronavirus. Although COVID-19 reduces respiration and blood oxygen and severely reduces movement and physical activity, low-intensity rehabilitation and breathing exercises along with medication can improve blood oxygen status, resting heart rate, blood pressure, and hand power status in patients and possibly speeding up the healing process. The results of the present study show that low-intensity exercise and breathing exercises in patients with COVID-19, whose disease severity is mild to moderate, can be performed safely under the supervision of their physicians to prevent the disease process.

## 1. Introduction

The epidemic of COVID-19, or acute respiratory syndrome, has spread rapidly worldwide, affecting the respiratory system and weakening the immune system, causing many deaths. The virus caused one of the biggest health crises in the world. It has been shown that viral infections, especially COVID-19, are increasing rapidly in overweight and obese people and that these patients are likely to experience a more severe course of the disease [1,2]. It has also been found that overweight and obese patients do not accurately report respiration and blood pressure. Research shows that lung volume decreases as people gain extra weight, which can cause impairments to their respiratory function [3]. Also, some of the other problems that occur with weight gain are blood pressure problems [4].

Furthermore, due to the fact that patients with COVID-19 suffer from severe motor loss, it is possible that they can suffer from muscle atrophy and physical impairment after medical treatments. Patients with COVID-19 have also been reported to experience severe fatigue and weakness in the muscles of the legs and arms when they develop the coronavirus disease [5]. This fatigue thus leads to limited movement and loss of muscle function [6]. Skeletal muscle is associated with the immune system and it has been shown that skeletal muscle regeneration works in the same direction as the immune system. In this process of atrophy, skeletal muscle can disrupt the immune system [7]. It has been reported that moderate-intensity exercise training can be an effective way to prevent severe cases of COVID-19 [8,9]. However, moderate physical exercise activities induce some cytokines and physiological responses that are probably not a good option during infection with COVID-19 [10]. It has been shown that low-intensity, short-term physical exercise does not cause significant changes in immunological and physiological responses [11]. In this regard, a study conducted on the rehabilitation of patients with COVID-19 found that low-intensity exercise could improve their respiratory and motor status. The mentioned study estimated the activity time in rehabilitation to be less than 20 min [12].

Rehabilitation exercise in patients may be effective in preventing muscle atrophy or at least its rapid progression. However, to the best of our knowledge, the effects of physical exercise during medical treatment on hospitalized COVID-19 patients have not been assessed in any study. Due to time and movement limitations in this type of patient, low-intensity exercise, as well as breathing exercises, could be effective solutions [3]. Therefore, this study was conducted to assess the effects of low-intensity, short-term physical exercise during hospitalized time on blood oxygen status, resting heart rate, blood pressure, and hand power status in an overweight patient with COVID-19.

## 2. Materials and Methods

### 2.1. Patient Introduction and Initial Conditions

**Figure 1:**
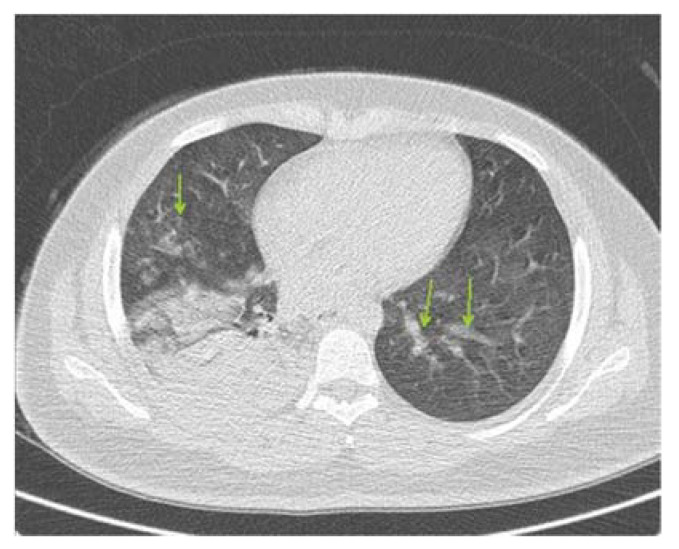
Lung involvement on the first day.

In the present case study, a 20-year-old woman with a body mass index (BMI) of 29 (overweight) from Rafsanjan, who did not have symptoms of diseases such as diabetes or cardiovascular disease, nor any allergies, and who was not using therapeutic pills, came to Hazrat Ali Ibn Abitaleb Hospital. The patient felt a weakness in her legs in the early hours of 2/3/2021, felt cold at 1 pm, and suffered from a severe migraine at night. The next day, the patient was shivering in the early hours of the morning and experienced a high fever at 4 pm. The patient had a high fever and chills in the night and could not rest until the morning because of this. The next day, the patient experienced initial shortness of breath, which changed when moving from lying down to squatting. The patient endured this condition until she went to the hospital due to severe shortness of breath, fever, and severe chills. When the patient went to the hospital and the initial tests were examined, the patient’s blood oxygen was 76. Therefore, the doctor ordered a CT scan of the lungs (Figure 1). After that, part of the right lung and part of the left lung were determined to be involved in the COVID-19 onset. As a result, the person was admitted to the women’s ward.

### 2.2. Ethical Considerations

Informed consent was obtained from the patient in this study and the exercise protocol was performed. All the steps and training protocols were explained accurately and in detail to the patient in a consent form, using simple and honest language, along with the standard treatments in the hospital, and finally the patient signed and approved the consent form. Ethical approval for this project was obtained from the Research Institute of Physical Education and Sports Sciences (Research Ethics Committee) with the number IR.SSRC.REC.1400.021.

### 2.3. Exercise Protocol

The exercise protocol lasted three days and included nine training sessions in the morning, evening, and night. The first session on the first day was performed in such a way that the patient sat on the bed with hands placed in the desired position next to the body, as if moving the forearm with a dumbbell or a barbell. In this movement, both hands hang next to the body and, through practice, the two hands are bent from the elbow and then returned to the original position. This movement was done in three sets with ten repetitions. The practitioner placed both hands on the patient’s wrist and the patient tried to bend her hands against the resistance of the practitioner. In the next exercise, the patient sat on the bed with the edge of the bed below the knees (as in the leg extension movement). In this movement, the practitioner put her two hands on the ankle and then the patient straightened her knee against the resistance of the practitioner’s hand and counted. After a pause of 1 s, she returned to the original position. This move was done in three sets with ten repetitions. Furthermore, the Borg scale (1 to 10) was used to evaluate the intensity of the exercise and the intensity of the exercise was controlled to be with the values of 1 to 3.

Finally, the patient’s last exercise included the isometric exercise of the upper torso and lower torso muscles. The patient was asked to contract the upper torso muscles for 10 s and then the lower torso muscles for 10 s. The patient was asked to perform this movement with her maximum power. The movement was performed in three sets with ten repetitions. The patient rested for 3 min between each set and for 3–5 min after each exercise, and the movement began with the announcement of readiness. Furthermore, because it was difficult for the patient to perform the breathing process without a mask, an oxygen mask was used in all stages of the exercise.

The second training session of the first day, which was performed in the evening, included breathing exercises. First, because it was difficult for the patient to breathe without an oxygen mask, breathing exercises were performed using an oxygen mask. Breathing exercises included deep breathing through the nose and, after a 3 s pause with air in the chest, breathing out of the mouth. This movement was performed in five sets. The exercise was stopped whenever the patient felt pain in the chest.

The third session of the first day was in the evening shift and was the same as the morning exercise protocol but with the difference that the repetition of resistance exercises was increased to 12 repetitions. Furthermore, the isometric contraction time increased to 12 s. Finally, the patient’s blood oxygen was checked and recorded after the last session of training on the first day. The training protocol of the second day was the same as the first day, with the difference that the number of repetitions in the morning exercises increased to 14 repetitions and the pause time for the breathing exercises in the evening to 5 s, while the night exercises were increased to 16 repetitions and the isometric contraction time to 16 s. Furthermore, on the second day, the patient’s blood oxygen was checked and recorded for the patient.

On the third day, in the morning, the number of repetitions was increased to 16 repetitions and the isometric contraction time to 16 s; in the evening shift, the pause time for the breathing exercises was increased to 7 s, the repetitions were increased to 18 repetitions, and the isometric contraction time to 18 s. Then, after resting, the patient’s blood oxygen was checked and recorded. Due to cardiorespiratory conditions and high fatigue in COVID-19 patients, the total training time in each shift was short (<20 min) [7]. Also, the patient’s symptoms were measured constantly and the exercise was stopped in consultation with the physician if symptoms were intensified or the patient felt discomfort. Further, to ensure the patient could breathe easily during the exercise, the oxygen mask was not removed during the movements.

The hand power status was measured with a dynamometer every day [7]. Finally, after three days of training, the patient’s blood oxygen increased. The researchers were sensitive to the patient’s symptoms and the exercise protocol was stopped whenever a physician judged it to be necessary. The patient showed gastrointestinal problems (heartburn) and the exercise protocol was stopped by the physician on the fourth day. The patient stated that on the days she was exercising, she slept comfortably.

### 2.4. Medications Taken by the Patient during Treatment

The patient used corticosteroids (dexamethasone 4 mL Bd), antibiotics (Vancomycin, Ceftriaxone, and Azithromycin), and antiviral drugs (Remdesivir) during the treatment.

## 3. Results

The results indicated that, after performing the exercise protocol, blood oxygen levels increased and heart rate and blood pressure returned to normal (Table 1). Patients with COVID-19 lose their muscle strength and this causes movement limitation; the exercise protocol described was able to increase hand strength in a patient suffering from these limitations. Leg muscle strength was not measured in this study but the patient stated that she could get out of the hospital bed and walk. She also said she felt fewer tremors and less weakness in her arms and legs. The results show that low-intensity, short-term exercises were able to prevent the patient from losing muscle strength. Furthermore, to be able to go to the bathroom from the hospital bed, the patient must remove the oxygen mask and move to the bathroom; on the first day, the patient felt very short of breath after doing this but, after exercising on the third day, the person felt less short of breath. This indicates the positive effect of breathing exercises.

## 4. Discussion

In the present study, a 20-year-old overweight patient with COVID-19 was able to respond positively after performing exercises. Upper respiratory tract infections (URTIs), such as coughs, colds, flu, sinusitis, tonsillitis, sore throats, and middle ear infections, are among the most common illnesses in all ages. Furthermore, it has been found that people who are obese or overweight are more prone to infectious diseases, especially respiratory diseases [1,2]. Coronavirus can severely affect the body’s immune system and many tissues, especially the lungs, by creating a cytokine storm in the body [10]. According to several reports, the lungs are exposed to the virus in people with COVID-19 and are completely deprived of movement in acute patients. However, in patients with moderate lung involvement, refereeing and short-term exercises can be a good solution.

The beneficial effects of exercise activities on various physiological responses, including the cardiovascular system, nervous system, metabolic system, etc., have been confirmed in many studies [13,14]. It is not possible to recommend moderate-intensity exercise activities to patients with COVID-19. However, it has been reported that one of the most effective intensities for increasing endorphins and reducing stress during exercise is moderate intensity, as regular exercise training can modulate some stress hormones and immunity [12]. Moderate exercise increases stress hormones, such as cortisol levels, which can suppress the immune function in a short time [15,16]. Moreover, people with COVID-19 are in a special condition, so low-intensity isometric exercises should be considered.

In the current study, the exercises included breathing exercises. The patient was able to perform breathing exercises using an oxygen mask. Therefore, it is recommended to have an oxygen mask when performing breathing exercises with patients with COVID-19.

In the study by Piquet et al. (2021) on rehabilitation time in patients with COVID-19, the researchers included two sessions of exercise in their rehabilitation program (with a researcher-made protocol). The first exercise program was intended to strengthen the overall movement related to body weight. In another part of the program, respiratory rehabilitation exercises including controlled diaphragmatic breathing were accompanied by breathing time and exhalation. Finally, the researchers stated that 10 days in the hospital using the exercise program could improve motor rehabilitation and functional outcomes [7]. The researchers also showed that mobility improved in these patients, which confirms the results of the present study.

With regard to the limitations of the research in the present study, the lack of a control group and the lack of control over drugs should be mentioned. Therefore, for future research proposals, researchers are advised to attend to these two limitations in their studies. Also, studies on the immunological and inflammatory changes of these patients during exercise could be considered in the future [17].

## 5. Conclusions

Our results proved that low-intensity physical exercises along with respiratory movements had no side effects for the patient during the treatment and probably accelerated the treatment process. Therefore, the applied exercise protocol can be considered for patients whose disease is not severe and who do not need to be admitted to the ICU. Also, the breathing exercises led by the practitioner were able to help improve the patient’s breathing condition [9]. Breathing and rehabilitation exercises must be done using an oxygen mask so that the patient does not suffer from hypoxia.

## Figures and Tables

**Table 1:** Results for the factors involved in COVID-19 after the training protocol.

Day	Blood Oxygen Status	Respiration	Resting Heart Rate	Blood Pressure (Resting)		Hand Power Status (Dynamometer)
		Number of Breaths Per Minute		Systolic	Diastolic	
First day	79	19	110	110	70	Two right hand attempts and one left hand attempt
Second day	84	18	95	111	60	Two right hand attempts and two left hand attempts
Third day	88	16	92	120	80	Three right hand attempts and three left hand attempts

## Data Availability

All data generated and analyzed during this study are included in this manuscript.
